# The fate of orally available antimicrobials

**DOI:** 10.1186/1753-6561-5-S6-P44

**Published:** 2011-06-29

**Authors:** B Catry, D Persoons, J Dewulf

**Affiliations:** 1 Healthcare associated infections (NSIH), Scientific Institute of Public Health, Brussels, Belgium; 2 Ghent University, Merelbeke, Belgium

## Introduction / objectives

Changing an antimicrobial treatment regimen has shown to influence the occurrence of antimicrobial resistance, with regimen consisting of the dose, the treatment interval, the duration of therapy, and the route of administration of the drug. For concentration dependent antimicrobials there is substantial evidence to encourage the use of a high dose, with regular treatment interval and short course to minimise the risk for the selection of resistant mutants. However, in contrast with these first three aspects of the antimicrobial treatment regimen, little attention is currently paid to the influence of route of administration for the probability of selection and spread of resistant strains.

## Methods

By comparing data from different animal species, the purpose is to explore the different routes of administration with regard to the stimulation of antimicrobial resistance.

## Results

A historical review on the emergence of resistance to beta-lactamas and tetracycline in *Staphylococcus aureus* and *Escherichia coli* in different animal species and humans will be provided. Data on commonly used treatment regimens for humans and animal will be compared and the potential impact on selection, co-selection and spread of resistance will be discussed escriptively. Special attention will be given to the difference in resistance development between topical, oral, and parenteral administration of antimicrobial agents.

## Conclusion

Attention to the route of administration might be an underestimated approach to mitigate the risk of selection for pathogens resistant to antimicrobials in both human and veterinary medicine.

## Disclosure of interest

None declared.

